# Phase II study of axitinib with doublet chemotherapy in patients with advanced squamous non–small-cell lung cancer

**DOI:** 10.1186/s12885-015-1350-6

**Published:** 2015-05-01

**Authors:** Igor M Bondarenko, Antonella Ingrosso, Paul Bycott, Sinil Kim, Cristina L Cebotaru

**Affiliations:** 1 Oncology Department, Dnepropetrovsk Medical Academy, City Multiple-Discipline Clinical Hospital, No. 4 31 Blizhnaya Street, Dnepropetrovsk, 49102 Ukraine; 2 Pfizer, Milan, Italy; 3 Pfizer Inc, San Diego, CA USA; 4 Prof. Dr. Ion Chiricută’ Institute of Oncology, Cluj-Napoca, Romania

**Keywords:** Non–small-cell lung cancer, Squamous cell, Axitinib, Anti-angiogenic treatment, Platinum-based chemotherapy, Phase II

## Abstract

**Background:**

Axitinib is an orally active and potent tyrosine kinase inhibitor of vascular endothelial growth factor receptors 1, 2 and 3. This phase II study assessed the efficacy and safety of axitinib combined with cisplatin/gemcitabine in chemotherapy-naïve patients with advanced/metastatic (stage IIIB/IV) squamous non–small-cell lung cancer (NSCLC).

**Methods:**

Axitinib (starting dose 5 mg twice daily [bid]; titrated up or down to 2–10 mg bid) was administered orally on a continuous schedule with cisplatin (80 mg/m^2^ intravenously [i.v.] every 3 weeks) and gemcitabine (1,250 mg/m^2^ i.v. on days 1 and 8 of each 3-week cycle), and was continued as monotherapy after completion of six cycles (maximum) of chemotherapy. The primary study endpoint was objective response rate, as defined by Response Evaluation Criteria in Solid Tumours.

**Results:**

Of the 38 patients treated, one (2.6%) patient achieved a complete response and 14 (36.8%) patients had a partial response; nine (23.7%) patients showed stable disease and three (7.9%) patients had disease progression. Median progression-free survival was 6.2 months, and median overall survival was 14.2 months. The estimated probability of survival at 12 months and 24 months was 63.2% and 30.8%, respectively. The most frequent grade ≥3 toxicities were neutropaenia and hypertension (13.2% each). Three (7.9%) patients experienced haemoptysis, of which one case (2.6%) was fatal.

**Conclusions:**

Treatment with the combination of axitinib and cisplatin/gemcitabine demonstrated anti-tumour activity in patients with advanced/metastatic squamous NSCLC and the fatal haemoptysis rate was low. However, without a reference arm (cisplatin/gemcitabine alone), it is not conclusive whether the combination is better than chemotherapy alone. This study was registered at ClinicalTrials.gov, registration # NCT00735904, on August 13, 2008.

**Electronic supplementary material:**

The online version of this article (doi:10.1186/s12885-015-1350-6) contains supplementary material, which is available to authorized users.

## Background

Non–small-cell lung cancer (NSCLC), a heterogeneous group of histologies that includes adenocarcinoma, squamous cell carcinoma and large cell carcinoma, accounts for approximately 85% of all lung cancers [1]. Patients with NSCLC typically present with locally advanced or metastatic disease at the time of diagnosis [2], and in these cases prognosis is poor, with a 5-year survival rate of less than 10% [3].

Platinum-based double-agent chemotherapy, which is standard first-line treatment for most patients with stage IIIB or IV NSCLC, is associated with an objective response rate of 17–38% and a median survival time of approximately 7 to 14 months [4-10]. Clinical evidence indicates that there are minimal differences in efficacy (objective response rate and overall survival) between the various platinum-based doublet regimens in the treatment of advanced NSCLC [11,12], and that the addition of a third cytotoxic agent increases toxicity but does not prolong survival [13]. Thus, it would appear that standard cytotoxic chemotherapy has reached a therapeutic plateau in advanced NSCLC [14]. As a consequence, current research is focused on addition of a molecularly targeted anti-angiogenic agent to double-agent chemotherapy [15,16].

Vascular endothelial growth factor (VEGF) is a key molecular target in the treatment of NSCLC [17,18], and the combination of VEGF-directed anti-angiogenic therapy with platinum-based doublet chemotherapy offers potential for improved outcomes in advanced NSCLC [15,19,20]. Bevacizumab, a recombinant humanised anti-VEGF monoclonal antibody with a plasma half-life of approximately 3 weeks [21], was the first anti-angiogenic agent to show a survival benefit when combined with standard cytotoxic chemotherapy in advanced non-squamous NSCLC, extending median overall survival beyond 12 months [22]. However, the use of bevacizumab is restricted by the risk of high-grade bleeding [23], including potentially fatal pulmonary haemorrhage [24], particularly among patients with squamous NSCLC [25]. Phase II evidence implicating squamous histology as a risk factor for bevacizumab-induced pulmonary haemorrhage [26] resulted in exclusion of patients with squamous NSCLC from subsequent clinical trials. Accordingly, bevacizumab is only approved for the treatment of non-squamous NSCLC [27]. Ramucirumab, a human monoclonal antibody targeting the VEGF receptor 2, has been recently approved by the US Food and Drug Administration as an add-on therapy for metastatic NSCLC. Ramucirumab plus docetaxel improved overall survival and progression-free survival compared with placebo plus docetaxel in patients with NSCLC whose disease progress after first-line treatment. Bleeding/haemorrhage events of any grade occurred more in the ramucirumab group compared with the placebo group; however, grade 3 or worse pulmonary haemorrhage did not differ between groups [28].

Axitinib (Inlyta®; Pfizer Inc, New York, NY, USA) is an orally active and potent small-molecule tyrosine kinase inhibitor that produces broad inhibition of the VEGF pathway by targeting all three VEGF receptor subtypes (VEGFR1, VEGFR2 and VEGFR3) and has a short plasma half-life of 2 to 5 hours [29]. Axitinib shows evidence of single-agent activity in advanced NSCLC [30], and acceptable toxicity, both as monotherapy [30,31] and when combined with chemotherapy, including cisplatin plus gemcitabine or carboplatin plus paclitaxel [32]. The objective of this phase II study was to assess the safety and efficacy of axitinib in combination with cisplatin and gemcitabine in chemotherapy-naïve patients with advanced/metastatic squamous NSCLC.

## Methods

### Patient selection

Patients aged ≥18 years with histologically or cytologically confirmed squamous NSCLC that was locally advanced (stage IIIB) with pleural effusion or metastatic (stage IV) or recurrent, and with measurable disease by Response Evaluation Criteria in Solid Tumours (RECIST) [33] were eligible for study inclusion. Patients were required to have an Eastern Cooperative Oncology Group performance status of 0 or 1, adequate renal and hepatic function and adequate bone marrow reserve (absolute neutrophil count ≥1,500 cells/μL, platelets ≥100,000 cells/μL). Patients were excluded from the study if they had received prior systemic therapy for stage IIIB/IV NSCLC (prior surgery or radiotherapy was permitted if completed ≥4 and ≥3 weeks, respectively, before enrolment) or prior anti-VEGF therapy; had lung lesions with cavitation or major blood vessel involvement, uncontrolled brain metastases or seizures, active malignancies other than NSCLC, gastrointestinal abnormalities or uncontrolled hypertension (systemic blood pressure [BP] >140/90 mm Hg); had experienced cardiovascular/cerebrovascular disease, bleeding diathesis or coagulopathy within 12 months of study entry or epileptic seizures or grade ≥3 haemoptysis/haemorrhage within 4 weeks of study entry; or had current or anticipated use of anti-coagulants or drugs known to be potent cytochrome P450 (CYP) 3A4 inhibitors or CYP1A2 or CYP3A4 inducers.

### Study design and treatment

This open-label, single-arm study was conducted at 10 centres in Poland, Romania, Ukraine and South Africa, from 17 December 2008 to 30 November 2011. The primary study endpoint was objective response rate, as defined by RECIST criteria; secondary endpoints included progression-free survival, overall survival, duration of response and safety. Progression-free survival was defined as the time from commencement of study medication to documentation of disease progression or death, whichever occurred first. Overall survival was defined as the time from commencement of study medication to death from any cause. Duration of response was defined as the time from first documentation of response to documentation of disease progression or death, whichever occurred first.

The study was approved by the independent ethics committee at each participating centre (see Additional file 1: Table S1) and was conducted in accordance with the Declaration of Helsinki and relevant International Conference on Harmonisation Good Clinical Practice guidelines. Written informed consent was obtained from all patients before entry into the study. The study is listed in the US National Institutes of Health ClinicalTrials.gov registry under the identifier NCT00735904 [34].

All patients received standard platinum-based doublet chemotherapy (cisplatin/gemcitabine) plus axitinib. Cisplatin (80 mg/m^2^ intravenous infusion) was administered on day 1 and gemcitabine (1,250 mg/m^2^ intravenous infusion) on days 1 and 8 of each 21-day cycle of chemotherapy, for a maximum of six cycles. Axitinib was administered orally on a continuous schedule at a starting dose of 5 mg twice daily (bid) and was continued as maintenance therapy after completion of chemotherapy until disease progression. The starting dose for axitinib (5 mg bid) was selected based on a phase I study of axitinib in combination with cisplatin/gemcitabine that indicated that a starting dose of 5-mg bid axitinib could be safely combined with standard doses of cisplatin/gemcitabine [32]. Axitinib could be up-titrated incrementally to 7 mg bid and then to a maximum dose of 10 mg bid if the patient showed no treatment-related grade ≥3 toxicity during 2 weeks of treatment with the existing dose. Dose up-titration was not permitted if the patient had a BP >150/90 mm Hg or was receiving anti-hypertensive therapy. Chemotherapy dose reductions were based on the maximum grade of haematological and non-haematological toxicities observed during the previous treatment cycle and on the day of initiation of the current dose. Subsequent chemotherapy dose re-escalation was permitted at the investigator’s discretion in the absence of grade ≥3 haematological and grade ≥2 non-haematological toxicities during the previous treatment cycle. Patients who discontinued chemotherapy due to toxicity were allowed to continue with axitinib monotherapy.

Stepwise reductions in axitinib dose from a starting dose of 5-mg bid to a minimum of 2-mg bid were mandated by the occurrence of treatment-related toxicities of grade ≥3 severity. In the event of marked hypertension (BP >160/105 mmHg), haemoptysis, proteinuria or grade 4 toxicity, axitinib treatment was interrupted until its resolution and restarted at a lower dose**.** Axitinib treatment was permanently discontinued if patients required axitinib dose reduction below 2-mg bid or dose interruption for >4 weeks, or if they showed evidence of nephrotic syndrome, lung cavitation or delayed (>1 week) resolution of haemoptysis. Patients who discontinued axitinib due to toxicity were allowed to continue with scheduled chemotherapy. Concomitant administration of potent CYP3A4/5 inhibitors and inducers, CYP1A2 and CYP2C8 substrates, non-steroidal anti-inflammatory drugs and coumarin-derivative anti-coagulants was discouraged during the study. However, if usage of a potent CYP3A4/5 inhibitor or inducer was necessary, agreement had to be obtained from the study sponsor.

### Study assessments

Tumour response was assessed by computed tomography (CT) or magnetic resonance imaging at baseline (within 28 days before commencing study treatment) and was repeated every 6 weeks during chemotherapy and every 8 weeks during axitinib maintenance therapy, using RECIST criteria. Complete and partial tumour responses were confirmed 4 weeks after first documentation. Physical examinations and serum chemistry and urinalysis tests were performed at baseline, and were repeated at 3- and 4-week intervals during chemotherapy and axitinib maintenance therapy, respectively. Haematology tests were performed at baseline, on days 1 and 8 of each cycle of chemotherapy and at 4-week intervals during axitinib maintenance therapy. Patients self-monitored their blood pressure bid during the study. Patients were followed-up at 2-month intervals after the final study visit to determine survival status. Patients who were not known to be deceased at the time of database closure were censored on the day when they were last known to be alive. Adverse events (AEs) were graded for severity using the National Cancer Institute Common Terminology Criteria for Adverse Events, version 3.0 [35].

### Statistical analyses

The study sample size (an accrual target of 36 patients) was based on a single-stage design to test the null hypothesis that the true objective response rate to treatment was ≤40% versus the alternative hypothesis that the true objective response rate was ≥60%, with type I and II error levels of 0.10 and 0.15, respectively. Efficacy and safety analyses were conducted on the intent-to-treat (ITT) population, which comprised all patients who received at least one dose of study medication. Descriptive statistics were used to summarise continuous variables, and frequency and percentages to summarise categorical variables. Two-sided 95% confidence intervals (CI) for objective response rates were calculated using the exact method based on the F distribution. Time-to-event endpoints (overall survival, progression-free survival and duration of response) were estimated using Kaplan-Meier survival analysis. The median time-to-event and 95% CI were determined for each endpoint.

## Results

### Patient characteristics and treatment

A total of 38 chemotherapy-naïve patients with advanced or metastatic squamous NSCLC were included in the study and received at least one dose of study medication (ITT population). Patients’ baseline demographics and clinical characteristics are summarised in Table 1. The majority of patients were white (97.4%), male (89.5%), had a history of smoking (86.8%) and had stage IV disease (86.8%). Eighteen (47%) patients received the maximum six cycles of combined gemcitabine/cisplatin chemotherapy. The median number of chemotherapy cycles started was 4 (range, 1–6). The median duration of axitinib therapy was 3.1 (range, 0.2–22) months, and 17 (44.7%) patients went on to receive axitinib maintenance therapy after chemotherapy***.*** The median dose of axitinib administered during the study was 10.0 mg/day (range, 6.2–19.6 mg/day). The majority (92.1%) of patients received concomitant medication during the study, most commonly ondansetron, dexamethasone or furosemide.

**Table 1:** **Baseline demographics and clinical characteristics of the ITT population**

Characteristic	Cisplatin + gemcitabine + axitinib (n = 38)
Age, years	
Mean (SD)	60.5 (7.1)
Median (range)	59.5 (47–73)
Gender	
Male	34 (89.5)
Female	4 (10.5)
Race	
White	37 (97.4)
Black	1 (2.6)
Smoking status	
Smoker	33 (86.8)
Non-smoker	5 (13.2)
Tumour histology	
Squamous cell carcinoma	38 (100)
Disease stage	
IIIB	5 (13.2)
IV	33 (86.8)
ECOG performance status	
0	12 (31.6)
1	26 (68.4)
Prior surgery	17 (44.7)
Bronchoscopy	11 (28.9)
Lymph node/pleural biopsy	6 (15.8)
Lobectomy	2 (5.2)
Thoracic wall resection	1 (2.6)

Values are n (%) unless otherwise noted. ECOG, Eastern Cooperative Oncology Group; ITT, intent-to-treat; SD, standard deviation.

### Efficacy

The investigator-assessed objective response rate (complete and partial responses) for the ITT population (n = 38) was 39.5% (95% CI, 24.0–56.6%). One (2.6%) patient had a confirmed complete response and 14 (36.8%) patients had a confirmed partial response on study medication; stable disease was reported in nine (23.7%) patients and disease progression in three (7.9%) patients (Table 2). Eight patients were ineligible for assessment of tumour response since the scheduled post-baseline CT scan was either unavailable or performed >28 days after the last study dose. Two further patients died before their first scheduled on-study tumour assessment (week 6 of chemotherapy) and one patient (excluded for protocol violation) did not undergo baseline tumour assessment. The median duration of response for patients with an objective tumour response (n = 15) was 5.8 months (95% CI, 4.7–7.2 months).

**Table 2:** Summary of tumour responses during the study period for the ITT population*

Tumour response, n (%)	Cisplatin + gemcitabine + axitinib, (n = 38)
Complete response	1 (2.6)
Partial response	14 (36.8)
Stable disease	9 (23.7)
Progressive disease	3 (7.9)
Indeterminate response^†^	8 (21.1)
Not assessed due to early death^‡^	2 (5.3)
Baseline status uncertain^§^	1 (2.6)
**Objective response** (complete + partial)	15 (39.5)

ITT = intent-to-treat.

*Study period comprised the treatment period plus 28-day follow-up period after the last dose of study drug.

^†^Imaging scans unavailable or performed >28 days after the last study dose.

^‡^Death occurring before the first scheduled tumour assessment.

^§^No baseline assessment performed.

**Figure 1:**
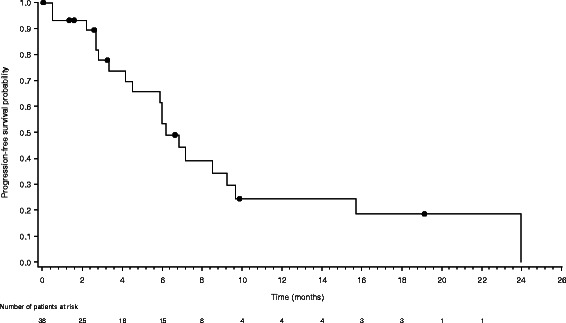
Kaplan-Meier curve of progression-free survival for the ITT population (n = 38). ITT, intent-to-treat.

**Figure 2:**
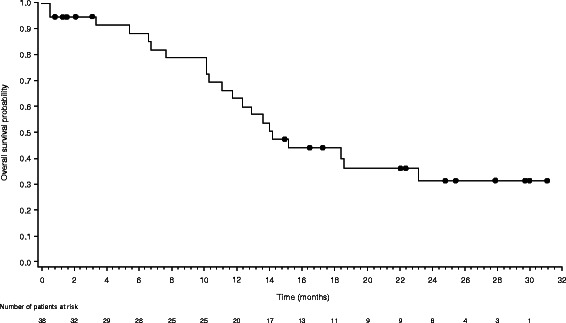
Kaplan-Meier curve of overall survival for the ITT population (n = 38). ITT, intent-to-treat.

Median progression-free survival after commencement of study medication was 6.2 months (95% CI, 4.5–9.3 months) (Figure 1). Median overall survival was 14.2 months (95% CI, 11.8–23.1 months) (Figure 2). The estimated probability of survival at 12 months and 24 months was 63.2% (95% CI, 44.7– 76.9%) and 30.8% (95% CI, 15.5–47.7%), respectively. In total, 21 (55.3%) patients died during the study (four patients during the study treatment period and 17 patients during follow-up).

### Safety

A total of 36 (94.7%) patients reported at least one AE (all-causality) of any grade, of which the most frequent were nausea (42.1%), anaemia (31.6%), vomiting (28.9%), hypertension (26.3%), neutropaenia (23.7%), weight loss (23.7%) and decreased appetite (21.1%) (Table 3). The most commonly reported grade ≥3 AEs were neutropaenia (13.2%), hypertension (13.2%), anaemia (7.9%) and fatigue (7.9%) (Table 3). Overall, 34 (89.5%) patients experienced treatment-related AEs (all grades). Fifteen (39.5%) patients experienced serious AEs while on treatment; the most frequent were anaemia, pneumonia, dehydration and disease progression (n = 2 each [5.3%]).

**Table 3:** Summary of all-causality adverse events occurring in >2 patients in the ITT population during the study period*

Adverse event	Cisplatin + gemcitabine + axitinib (n = 38)			
	All grades		Grade ≥3	
	No.	%	No.	%
Nausea	16	42.1		
Anaemia	12	31.6	3	7.9
Vomiting	11	28.9	2	5.3
Hypertension	10	26.3	5	13.2
Neutropaenia	9	23.7	5	13.2
Weight loss	9	23.7	0	0
Appetite loss	8	21.1	0	0
Fatigue	7	18.4	3	7.9
Asthenia	6	15.8	0	0
Leukopaenia	5	13.2	2	5.3
Diarrhoea	5	13.2	0	0
Reduced creatinine clearance	5	13.2	0	0
Alopecia	5	13.2	0	0
Thrombocytopaenia	4	10.5	0	0
Chest pain	4	10.5	0	0
Toxic nephropathy	4	10.5	0	0
Cough	4	10.5	0	0
Dyspnoea	4	10.5	0	0
Rash	4	10.5	0	0
Pulmonary cavitation	3	7.9	1	2.6
Haemoptysis	3	7.9	1	2.6

ITT = intent-to-treat.

*Study period comprised the treatment period plus 28-day follow-up period after the last dose of study drug.

Fatal pulmonary haemorrhage is one of the major safety concerns in patients receiving anti-angiogenic therapy for squamous NSCLC. Three (7.9%) patients had haemoptysis, including two patients with grade 1 severity and one patient with grade 5 severity. The latter patient developed massive haemoptysis on day 16 of the first treatment cycle and died later that day from NSCLC; there was no prior evidence of tumour cavitation on CT scan or X-ray, and no obvious risk of haemoptysis in the patient’s medical history. One case of grade 1 haemoptysis was considered to be related to axitinib treatment. Three (7.9%) patients had elevated systolic BP (≥160 mm Hg) during the study, whereas none had elevated diastolic BP (≥105 mm Hg). Clinically significant laboratory abnormalities included elevated alanine aminotransferase (5.3%) and aspartate aminotransferase (2.6%), increased blood creatinine (5.3%) and reduced renal creatinine clearance (13.2%).

Treatment-emergent AEs resulted in at least one dose interruption in 13 (34.2%) patients, and dose reduction in five (13.2%) patients. The most frequent reasons for dose interruption were vomiting and anaemia (n = 3 each [7.9%]) and nausea, fatigue, dyspnoea and hypertension (n = 2 each [5.3%]), whereas the most frequent reason for dose reduction was hypertension (n = 2 [5.3%]). Overall, eight (21.1%) patients discontinued treatment due to AEs during the study, with four (10.5%) patients discontinuing because of drug-related toxicity (reduced renal creatinine clearance, pulmonary cavitation, pulmonary embolism and hypertension, n = 1 each). Four (10.5%) patients died while on treatment (disease progression, n = 3; cerebrovascular accident and multiple organ failure, n = 1).

## Discussion

This single-arm phase II study demonstrated that the combination of axitinib with cisplatin/gemcitabine has anti-tumour activity in advanced/metastatic squamous NSCLC, as reflected in an objective response rate of 39.5% (95% CI, 24.0–56.6%), a median overall survival of 14.2 months (95% CI, 11.8–23.1 months) and a 1-year survival rate of 63.2% (95% CI, 44.7–76.9%). The confirmed objective response rate (based on investigator assessment) was, however, only marginally higher than that previously reported with doublet chemotherapy (17% to 38%) in advanced NSCLC [4-11] and, accordingly, the null hypothesis that the true response rate is ≤40% was not rejected. The median overall survival of 14.2 months with the combination of axitinib plus cisplatin/gemcitabine was appreciably higher than most previously reported results for the doublet chemotherapy in NSCLC, where median overall survival ranged between 7.0 and 12.9 months [6-10,36] and was similar to a previous study where the median overall survival was 14.0 months with the doublet chemotherapy [8]. The 1-year survival rate of 63.2% appear to be only marginally higher than the 55.9% [6] and 59.6%% [8] reported previously for cisplatin/gemcitabine treatment.

The toxicities caused by the combination of axitinib with standard chemotherapy were manageable in this selected patient population. The pattern and frequency of AEs observed during the study — predominantly nausea, anaemia, vomiting, hypertension, neutropaenia, weight loss, decreased appetite and fatigue — were consistent with the oncology setting and reflect the overall poor health of patients with advanced/metastatic NSCLC. Of note, life-threatening pulmonary haemorrhage, which is a particular safety concern with anti-angiogenic agents in squamous NSCLC [25], was detected in one (2.6%) patient who experienced fatal grade 5 haemoptysis during the first treatment cycle. No risk factors for haemoptysis were identified in the patient’s medical history. Although the investigator on site considered the fatal event not to be related to the study medications but to NSCLC, it is impossible to rule out the relationship of axitinib to the development of haemoptysis especially given the known risk of haemoptysis with VEGF inhibitors. In contrast, a randomised phase II study of bevacizumab, carboplatin and paclitaxel combination therapy in advanced NSCLC reported four cases of life-threatening pulmonary haemorrhage among 13 (30.8%) patients with squamous histology [26]. The long plasma half-life of bevacizumab may have contributed to the severity of pulmonary haemorrhage, since its anti-angiogenic effect cannot be reversed rapidly.

Single-agent tyrosine kinase inhibitors, including axitinib, have proved to be generally well tolerated in patients with NSCLC [30,37-40]. The most common treatment-related AEs reported with axitinib monotherapy in NSCLC include fatigue, anorexia, diarrhoea and nausea, and these can be managed with dose modification and/or supportive treatment [30]. Although hypothyroidism has been linked with fatigue as a class effect of VEGFR-targeted therapy [41], we found no evidence of an increase in thyroid-stimulating hormone levels during the study. Hypertension, which is commonly observed with anti-angiogenic agents [42], occurred in one in four axitinib-treated patients, but was managed through use of anti-hypertensive treatment or axitinib dose reduction.

Due to their ability to inhibit multiple angiogenesis pathways, tyrosine kinase inhibitors offer the potential for improved efficacy and decreased secondary resistance [43]. In this study, the combination of axitinib with cisplatin/gemcitabine provided similar response rate and median overall survival when compared with corresponding historical data for cisplatin/gemcitabine chemotherapy alone. These results are consistent with previously reported studies of combined chemotherapy with axitinib [44] and other angiogenic tyrosine kinase inhibitors in NSCLC [45-47]. A recent randomised phase II trial of axitinib in combination with pemetrexed/cisplatin in patients with non-squamous NSCLC showed that the addition of axitinib resulted in numerically higher objective response rate but did not significantly improve median progression-free survival or median overall survival compared with chemotherapy alone [44]. The Motesanib NSCLC Efficacy and Tolerability (MONET1) study, which assessed the effect of adding motesanib, a small-molecule targeted antagonist of VEGFR-1, 2 and 3, to doublet chemotherapy (carboplatin and paclitaxel) compared with chemotherapy alone for first-line therapy of non-squamous NSCLC, reported a significant improvement in tumour response rate (40% vs. 26%, respectively), but no benefit in overall survival (median 13 vs. 11 months, respectively) [47]. Likewise, two randomised phase III trials, Evaluation of Sorafenib, Carboplatin and Paclitaxel Efficacy (ESCAPE) and NSCLC Research Experience Using Sorafenib (NEXUS), found no significant survival benefit from addition of sorafenib to platinum-based chemotherapy in unresectable stage IIIb/IV NSCLC [45,46]. Indeed, in patients with squamous histology, sorafenib appeared to reduce median overall survival (8.9 vs. 13.7 months, sorafenib plus chemotherapy vs. chemotherapy alone, respectively); however, it should be noted that the overall survival time of patients receiving chemotherapy alone in the ESCAPE trial (median 13.7 months) was much greater than expected [46]. Similarly, in the phase III Iressa NSCLC Trial Assessing Combination Treatment (INTACT) 1 and 2 trials, no overall survival benefit was obtained from addition of the epidermal growth factor tyrosine kinase inhibitor gefitinib to platinum-based chemotherapy in chemotherapy-naïve patients with advanced/metastatic NSCLC [48,49]. The addition of the epidermal growth factor receptor (EGFR)-directed monoclonal antibody cetuximab to platinum-based chemotherapy has produced mixed results in advanced NSCLC, with a significant improvement in overall survival being reported in one study [50] but not replicated in another study [51].

Taken together, these results may suggest that the combination of chemotherapy plus multi-targeted anti-angiogenic tyrosine kinase inhibitor therapy may not be advantageous over chemotherapy alone in terms of overall response rate, progression-free survival and overall survival in advanced NSCLC.

The results of this study should be considered with respect to its limitations. This was a single-arm study with a small number of patients. With the lack of a reference arm (cisplatin/gemcitabine alone), the results were compared with previous studies and these historical cross-trial comparisons should be interpreted with caution because of potential differences in each study, including the inclusion/exclusion criteria, diagnosis and staging and other differences [52].

## Conclusions

In conclusion, the combination of axitinib with cisplatin plus gemcitabine demonstrated anti-tumour activity in patients with advanced/metastatic squamous NSCLC. Further, the safety profile was consistent with the oncology setting and reflects the overall poor health of these patients. Severe pulmonary haemorrhage, a potential life-threatening toxicity associated with anti-angiogenic treatment of squamous NSCLC, occurred in one patient. The study findings provide preliminary indications that median overall survival in advanced NSCLC can be extended beyond the 12-month threshold. However, due to the absence of a reference arm of cisplatin/gemcitabine alone in this study, it is not conclusive whether the combined treatment is better than chemotherapy alone.

## Additional file

Additional file 1: Table S1. List study centres and corresponding ethics committees or institutional review boards.

## Abbreviations

AE: Adverse event
bid: Twice daily
BP: Blood pressure
CI: Confidence interval
CT: Computed tomography
CYP: Cytochrome P450
ECOG: Eastern Cooperative Oncology Group
ITT: Intent to treat
NSCLC: Non–small-cell lung cancer
RECIST: Response Evaluation Criteria in Solid Tumours
SD: Standard deviation
VEGF: Vascular endothelial growth factor
VEGFR: Vascular endothelial growth factor receptor
