# Supplementary material for: Phase II study of axitinib with doublet chemotherapy in patients with advanced squamous non–small-cell lung cancer
Source: BMC Cancer. 2015 May 1;15:339. doi: 10.1186/s12885-015-1350-6 (PMC4424571; doi:10.1186/s12885-015-1350-6)
Supplement: Additional file 1: Table S1. — List study centres and corresponding ethics committees or institutional review boards. [file 12885_2015_1350_MOESM1_ESM.doc]

**Additional file 1: Table S**1. List study centres and corresponding ethics committees or institutional review boards.

| **Center Number** | **Ethics Committee or Institutional Review Board** | **City, State/Province, Postal Code, Country** |
| --- | --- | --- |
| 1004 | Komisja Bioetyczna Slaskiej Izby  Lekarskiej w Katowicach | ul. Grazynskiego 49A  Katowice, 40-126  POLAND |
| 1005 | Komisja Bioetyczna Slaskiej Izby  Lekarskiej w Katowicach | ul. Grazynskiego 49A  Katowice, 40-126  POLAND |
| 1009 | Academia de Stiinte  Medicale,Comisia Nationala de Etica | pentru Studiul Clinic al Medicamentului  Str. Av. Sanatescu, nr. 48, sector 1, Bucuresti, 011478  ROMANIA |
| 1010 | Academia de Stiinte  Medicale,Comisia Nationala de Etica | pentru Studiul Clinic al Medicamentului  Str. Av. Sanatescu nr. 48, Sector 1, Bucuresti, 011478  ROMANIA |
| 1013 | Academia de Stiinte  Medicale,Comisia Nationala de Etica | pentru Studiul Clinic al Medicamentului  Str. Av. Sanatescu nr. 48, Sector 1, Bucuresti, 011478  ROMANIA |
| 1002 | University of the Witwatersrand  Human Research Ethics Committee (Medical) | 8 Blackwood Avenue  Parktown  Johannesburg, 2193  SOUTH AFRICA |
| 1006 | Central Committee for Ethics Issues  of Ministry of Health of Ukraine | 5, Narodnogo opolchennya Str.  Kyiv, 03680  UKRAINE |
|  | Committee for Ethics Issues at  Dnipropetrovsk City Multiple-  Discipline Clinical Hospital #4 | 31, Blyzhnya Street  Dnipropetrovsk, 49102  UKRAINE |
|  | Committy for Bioethical Issues of  Dnipropetrovsk State Medical  Academy  Committy for Bioethical Issues of  Dnipropetrovsk State Medical  Academy | 9, Dzerzhinskogo Str.  Dnipropetrovsk, 49044  UKRAINE |
| 1007 | Central Committee for Ethics Issues  of Ministry of Health of Ukraine | 5, Narodnogo opolchennya Street  Kyiv, 03680  UKRAINE |
|  | Local Ethics Committee of Donetsk  Regional Antitumoral Center | 2a, Polotska Street  Donetsk, 83092  UKRAINE |
| 1008 | Central Committee for Ethics Issues  of Ministry of Health of Ukraine | 5, Narodnogo opolchennya Str.  Kyiv, 03680  UKRAINE |
|  | Local Ethic Committee Lviv State  Oncologic Regional Treatment and  Diagnostic Center | Lviv State Oncologic Regional  Treatment and Diagnostic Center  2A, Ja. Gashek Street  Lviv, 79031  UKRAINE |
| 1011 | Central Ethics Committee Ministry  of Health of Ukraine | 5, Narodnogo opolchennya Str.  Kyiv, 03680  UKRAINE |
|  | Local Committee for Ethics Issues of  Kyiv Regional Oncologic Dispensary | 1 Baggoutivska Street  Kyiv, 04107  UKRAINE |
